# Adipocyte Dysfunction in a Mouse Model of Polycystic Ovary Syndrome (PCOS): Evidence of Adipocyte Hypertrophy and Tissue-Specific Inflammation

**DOI:** 10.1371/journal.pone.0048643

**Published:** 2012-10-31

**Authors:** Joseph S. Marino, Jeffrey Iler, Abigail R. Dowling, Streamson Chua, Jens C. Bruning, Roberto Coppari, Jennifer W. Hill

**Affiliations:** 1 Department of Physiology and Pharmacology, Center for Diabetes and Endocrine Research, University of Toledo Medical Center, Toledo, Ohio, United States of America; 2 Department of Obstetrics-Gynecology, University of Toledo Medical Center, Toledo, Ohio, United States of America; 3 Departments of Medicine and Neuroscience, Albert Einstein College of Medicine, New York, New York, United States of America; 4 Department of Mouse Genetics and Metabolism, Institute for Genetics, Cologne Excellence Cluster for Cellular Stress Responses in Aging Associated Diseases, and Center for Molecular Medicine Cologne, 2nd Department for Internal Medicine, University of Cologne, and Max Planck Institute for the Biology of Aging, Cologne, Germany; 5 Departments of Internal Medicine, Division of Hypothalamic Research, Pharmacology, and Psychiatry, The University of Texas Southwestern Medical Center, Dallas, Texas, United States of America; Imperial College London, United Kingdom

## Abstract

Clinical research shows an association between polycystic ovary syndrome (PCOS) and chronic inflammation, a pathological state thought to contribute to insulin resistance. The underlying pathways, however, have not been defined. The purpose of this study was to characterize the inflammatory state of a novel mouse model of PCOS. Female mice lacking leptin and insulin receptors in pro-opiomelanocortin neurons (IR/LepR^POMC^ mice) and littermate controls were evaluated for estrous cyclicity, ovarian and adipose tissue morphology, and body composition by QMR and CT scan. Tissue-specific macrophage infiltration and cytokine mRNA expression were measured, as well as circulating cytokine levels. Finally, glucose regulation during pregnancy was evaluated as a measure of risk for diabetes development. Forty-five percent of IR/LepR^POMC^ mice showed reduced or absent ovulation. IR/LepR^POMC^ mice also had increased fat mass and adipocyte hypertrophy. These traits accompanied elevations in macrophage accumulation and inflammatory cytokine production in perigonadal adipose tissue, liver, and ovary. These mice also exhibited gestational hyperglycemia as predicted. This report is the first to show the presence of inflammation in IR/LepR^POMC^ mice, which develop a PCOS-like phenotype. Thus, IR/LepR^POMC^ mice may serve as a new mouse model to clarify the involvement of adipose and liver tissue in the pathogenesis and etiology of PCOS, allowing more targeted research on the development of PCOS and potential therapeutic interventions.

## Introduction

Polycystic ovary syndrome (PCOS) is a common endocrine disorder in women of reproductive age, affecting 5–10% of this group [1]. Besides infertility, PCOS is associated with a range of adverse health outcomes, including type 2 diabetes and cardiovascular disease [2], [3], [4]. Many PCOS patients are obese or show increased abdominal adiposity, which may worsen the severity of the syndrome and its complications [5], [6], [7], [8].

In the general population, obesity contributes to adipose tissue dysfunction. Large adipocytes or limited adipocyte differentiation is associated with insulin resistance and diabetes risk [9], [10]. The adipose tissue of obese individuals is often characterized by hypertrophic adipocytes, a proinflammatory gene expression profile, and dysregulated secretion of adipose-specific proteins and cytokines [6], [11]. This pro-inflammatory response, possibly resulting from hypoxia in the adipocyte [12], [13], [14], leads to an increase in the recruitment of macrophages and other immune cells [15], [16], [17]. It is likely that the development of adipose inflammation and dysfunction is a process that begins early in the expansion of adipose depots. Indeed, adipose expression of macrophage chemoattractant proteins rises significantly following only 1 week on a high fat diet [18].

Obesity-related inflammation also results in impaired liver-mediated regulation of glycemia and reduced skeletal muscle insulin sensitivity [19], [20]. Cytokines and macrophage-secreted factors activate inflammatory pathways in cells that sense insulin. These pathways lead to the phosphorylation of insulin receptor substrate (IRS) proteins and insulin receptors, interfering with normal insulin action and creating a state of cellular insulin resistance [21]. Although the normal mode of cytokine-mediated insulin resistance involves local paracrine effects, elevated blood levels of TNFα, interleukin (IL)-6, and IL-1β have been reported in obese insulin-resistant states, raising the possibility that tissue cytokines that pass into the circulation can impair insulin sensitivity in distal tissues [19], [22]. Indeed, inflammatory mediators appear to induce insulin resistance locally in fat and liver, but also systemically in skeletal muscle [23], [24]. In addition, increased proinflammatory cytokine levels in the bloodstream may promote atherogenesis and CVD [25].

In both lean and obese PCOS patients, circulating markers of low-grade inflammation are elevated, suggesting that inflammation can promote insulin resistance, atherosclerosis, and other pathologies associated with PCOS [26], [27], [28], [29]. In particular, PCOS patients have elevated circulating IL-18, TNF*α*, IL-6, high sensitivity C-reactive protein (hs-CRP), monocytes, T cells, B cells, and neutrophils compared with body mass index matched controls [26], [30], [31]. In contrast, others have found that the accumulation of adipose tissue rather than the presence of PCOS is the primary cause of the pro-inflammatory response [31]. Therefore, additional investigation is necessary to identify the specific contribution of PCOS to the development of the low-grade inflammatory response identified in these patients. The detection of a preclinical inflammatory process would be a useful prognostic and therapeutic monitoring tool for PCOS patients at risk for the development of diabetes or CVD. The invasive nature of tests for tissue-specific inflammation makes the use of an animal model to identify markers for early detection highly advantageous. Rodent models of the reproductive and metabolic disturbances of PCOS can provide an invaluable means to investigate the etiology and perhaps promote research into the prevention and treatment of PCOS and its co-morbidities.

The relevance of current animal models of PCOS that involve the administration of androgens or other steroid hormones to the etiology of PCOS in humans is uncertain [32], [33]. We have recently shown that specific genetic deletion of insulin receptors (IR) and leptin receptors (LepR) from pro-opiomelanocortin (POMC) neurons reproduces many features of PCOS. POMC neurons modulate food intake, energy use, and hepatic glucose production [34], [35], [36]. These IR/LepR^POMC^ mice have normal GnRH gene expression, and normal estradiol and prolactin levels [37]. However, by four months old and before the onset of obesity, they show reduced litter production, reduced litter sizes, and lengthened reproductive cycles [37]. In addition, their serum testosterone levels are significantly elevated, with increased expression of ovarian 3β-HSD [37], the enzyme that produces androstenedione. IR/LepR^POMC^ mice also show significantly increased basal insulin levels, reduced glucose tolerance, overall insulin resistance, and profound hepatic insulin resistance resulting in increased glucose production [37]. Although these mice do not overeat, they have a decreased metabolic rate [37]. As in PCOS [38], [39], [40], their insulin resistance is out of proportion to their body weight. The body weights of females do not diverge from controls until 6 months old when they display increased fat deposition [37]. Therefore, like PCOS patients, these mice display both reproductive and metabolic abnormalities.

The primary aim of the present study was to determine whether this new mouse model of PCOS has chronic inflammation. We hypothesized that IR/LepR^POMC^ mice would be predisposed to adipocyte dysfunction along with PCOS-like impairments in fertility before obesity onset, allowing assessment of adipocyte function with a reduced number of confounding factors. After verifying sensitivity of the HPG axis to metabolic status, we assessed the following: 1) ovarian morphology, 2) fat distribution and adipocyte size, 3) adipose tissue macrophage infiltration and 4) circulating cytokine levels and cytokine mRNA expression in adipose tissue, liver, and ovary. Because impaired adipose tissue function in women with PCOS could promote insulin resistance and susceptibility to diabetes [41], we also investigated the development of gestational diabetes in these mice.

## Materials and Methods

### Experimental animals

Mice lacking leptin receptors (LepRs) and insulin receptors (IRs) in pro-opiomelanocortin (POMC) cells (IR/LepR^POMC^ mice) have been previously described [37]. Separate cohorts of animals were generated for each of the experiments outlined below. Study animals were derived from crosses between 1) males homozygous for the floxed IR and LepR carrying the POMC-cre allele and 2) females homozygous for each floxed receptor. Experimental mice were compared with littermate controls carrying only the floxed alleles (IR/Lepr^flox^ mice). All mice were on a mixed C57BL/6J;129S6/SvEv background.

All animal care and procedures were approved by the University of Toledo Medical School or UT Southwestern Medical Center Institutional Animal Care and Use Committees. Mice were housed in a temperature-controlled environment in groups of two to four at 22C–24C using a 12 hr light/12 hr dark cycle with standard chow (4% fat mouse/rat diet #7001, Harlan-Teklad, Madison, WI) and water provided *ad libitum* unless noted. Mice were killed by CO^2^ narcosis unless otherwise specified.

### Examination of Ovarian Morphology

Ovaries were removed at autopsy at 6 months old and fixed for 72 hours in 4% paraformaldehyde. The tissues were then embedded in paraffin, cut into 20-micron sections on a sliding microtome, and stained with hematoxylin/eosin. The numbers of corpora lutea and follicles of various stages present in slices from each ovary were tabulated for each of the genotypes. Follicles were labeled as primordial (surrounded by a single layer of flat, squamous granulosa cells), pre-antral (surrounded by up to Five layers of cuboid granulose cells), early antral (small antrum visible), or preovulatory (largest size with well-defined corona radiata surrounding the zona pellucida). Follicles in which the oocyte was not visible were not counted to avoid duplication. All sections were examined by a single blinded observer. Average counts were calculated by dividing total follicles counted by the number of sections examined per ovary. Only high-quality sections were quantified. An average of 4.4 sections were counted per ovary.

### PCNA Immunohistochemistry

Ovarian sections were prepared as described above. Sections were permeabilized with 0.5% triton X-100 in tris buffered saline (TBS) for 10 followed by by 2 washes with TBS. Sections were then incubated with 0.3% hydrogen peroxide in 70% methanol made in TBS for 15 minutes to quench endogenous peroxidase activity. Following 2 washes with TBS, sections were incubated in blocking buffer (3% donkey serum, 0.5% triton X-100 in TBS) for 30 minutes at room temperature. Sections were washed and incubated with mouse-anti PCNA (cat.# MCA1558; AbD Serotec) 1∶100 in TBS for 2 hours at room temperature. Sections were washed 3 times and incubated with biotinylated sheep anti-mouse IgG (cat.# AAC108; AbD Serotec) 1∶50 in TBS for 30 minutes at room temperature. Sections were washed with TBS and incubated for 30 minutes with horseradish peroxidase avidin D, 1∶1000 in TBS. Following 2 final washes with TBS, sections were developed using AEC (Vector Labs).

### Examination of Impact of Fasting on Estrous Cyclicity

Vaginal cytology was examined for at least 3 weeks to establish average estrous cycle lengths of female IR/LepR^POMC^ mice and littermate controls. Estrous cycles were monitored by daily inspection of vaginal lavage as described by Bingel and Schwartz [42]. Estrous cycle length was defined as the number of days from one proestrous stage to another. The impact of fasting was then determined at 6 months of age. Food was removed during diestrus, an hour before lights out (1900 h) and returned 48 hours later. Vaginal cytology was monitored until one full cycle occurred (P-E-D-M).

### Examination of Adipose Depots

Four-month-old female IR/LepR^POMC^ mice and littermate control mice were examined for these studies. Body weight was measured on an Ohaus Voyager precision mouse scale. Body composition was measured using quantitative magnetic resonance (QMR) (Bruker's Minispec MQ10, Houston, TX). Fat volume in subcutaneous and intra-abdominal depots was measured by CT scan. For in vivo scans, mice were anesthetized by 1% isoflurane inhalation and positioned in the mouse bed. The whole body (base of the skull, as the spinal canal begins to widen and the distal end of the tibia) of each mouse was scanned at an isotropic voxel size of 93 µm (80 kV, 450 µA and 100 ms integration time) using the eXplore Locus micro-CT scanner (GE Health Care). Each scan took about 10 minutes, and the mice showed no sign of discomfort during the procedure. The legs and head were not scanned. Scan energy and voxel size (scanning increment) was decided by the requirements of scanning time and tissue detail, and on minimizing exposure to radiation. Based on the scan parameters, the estimated radiation exposure was 4 rad (0.04 Gy) for each scan. Three-dimensional images were reconstructed from two-dimensional gray-scale image slices and were visualized using Microview Software (GE Medical SystDensity). Values for soft tissue and bone were calibrated from a phantom containing air, water and hydroxyl apatite rod (GE Health Care). Fat analysis was conducted using microview software with an advanced fat analysis tool. For calculation of adipose tissue volume, a ROI (region of interest) was drawn around the body of the animal, and a histogram was created on this selected region. The separation of fat regions was calculated from the appropriate gray scale value (upper threshold −165 and lower threshold −360) on the histogram. The abdominal muscular wall was used as the differentiation line to separate intra-abdominal adipose tissue from subcutaneous adipose tissue. The contour lines were drawn around the viscera and a 3-dimensional ROI was generated. The intra-abdominal fat was measured from the histogram of these segmented viscera using the same thresholds. Subcutaneous fat was calculated by subtracting intra-abdominal fat from the total body fat.

At autopsy, perigonadal adipose tissue was rapidly removed from each mouse. The tissues were immediately fixed in 10% neutral buffered formalin, embedded in paraffin, and processed for histological analysis. All the tissues were sectioned (5 µm thick) and routinely stained using hematoxylin and eosin. A morphometric analysis was performed using Nikon NIS-Elements BR3.1 software (Tokyo, Japan). The adipocyte area was calculated by capturing bright-field images and measuring an area containing 50 cells for each mouse, and the average area per cell was calculated. Cell counting and sizing were performed by blinded operators to avoid any biases.

### Examination of Inflammatory Markers

Immunohistochemistry was performed on perigonadal adipose tissue collected from 4 month old IR/LepR^POMC^ and IR/LepR (control) mice. Briefly, tissue was fixed overnight in 10% formalin and paraffin embedded before sectioning. Following 30 minutes of blocking, sections were incubated overnight at 4°C with rat anti-mouse F4/80 (abCam) at a dilution of 1∶50. Alexa Fluor 488 (Invitrogen) secondary was used to visualize F4/80 positive cells, and nuclei were labeled with DAPI (Vector Labs). For each set of slides immunostained for F4/80, one cross section from an IR/LepR^POMC^ mouse was used as an antibody control to test specificity of the primary antibody.

For QPCR analysis, RNA was isolated from perigonadal fat, liver, and ovary from 4 month old IR/LepR^POMC^ and IR/LepR (control) mice using Qiagen Rneasy kit (Qiagen). cDNA was reverse transcribed using Applied Biosystems high capacity RT kit (Applied Biosystems). F4/80, and cytokine gene expression was measured using a Step One Plus (Applied Biosystems) system and expression levels were normalized to 18S (adipose) or GAPDH (ovary and liver). Changes in mRNA expression were calculated using 2^(−ΔΔct)^ and expressed as a fold change compared with controls (IR/LepR). Sequences for primers used from 5′ to 3′ are: F4/80 forward CTTTGGCTATGGGCTTCCAGTC and reverse GCAAGGAGGACAGAGTTTATCGTG, CD11c forward CTGGATAGCCTTTCTTCTGCTG and reverse GCACACTGTGCCGAACTCA, IL-1β forward CAACCAACAAGTGATATTCTCCATG and reverse GATCCACACTCTCCAGCTGCA, IL-6 forward CTGCAAGAGCTTCCATCCAGTT and reverse GAAGTAGGGAAGGCCGTGG,18s forward TTGACGGAAGGGCACCACCAG and reverse GCACCACCACCCACGGAATCG and GAPDH forward ATGTTTGTGATGGGTGTGAA and reverse ATGCCAAAGTTGTCATGGAT.

### Serum analysis

Blood from randomly cycling adult female mice (4 months old) was collected transcardially at 3 hours after lights on to avoid any potential influence of increased gonadal steroids in the afternoon of certain phases of the estrous cycle. Serum was analyzed as follows. Triglycerides were analyzed with commercially available reagents (Pointe Scientific, Inc.). Total cholesterol, HDL, and LDL/VLDL were analyzed with a commercially available kit (BioAssay Systems), with a lower limit of detection of 5 mg/dl. C-reactive protein (Alpco) analysis was performed according to the manufacturer's protocol with a lower limit of detection of 0.39 ng/ml. Cytokine concentrations were analyzed using a Bio-Plex cytokine array (Bio-Rad) and measured using the Bio-Plex Plate Reader (Bio-Rad) with a lower limit of detection of 0.2 pg/ml. Serum free fatty acids (BioVission) were analyzed according to the manufactures protocol with a lower limit of detection of 2 µM. For all serum analyses, samples were run in at least duplicate and diluted to fit in the respective standard curves.

### Examination of Gestational Glucose Regulation

Basal glucose levels were measured in experimental and control females at 3 months of age. Glucose in tail blood was assayed using a mouse-specific glucometer (AlphaTRAK, Abbott, IL), with non-fasted measurements taken between 8 and 10 a.m.

Another cohort of experimental and control females were paired at 3.5 to 4 months of age with male control mice known to be successful breeders. Females were weighed and monitored for vaginal plugs each morning by 2 hours after lights on. The male was removed after a plug was found, and the female was weighed daily to verify pregnancy-related weight gain. Mice that became pregnant within 2 weeks were used for these studies. All mice delivered on their expected due dates.

Fasted glucose levels were measured before pairing and on gestational day 12 and 15. For glucose tolerance tests (GTTs), 6 h-fasted mice were injected with i.p. D-glucose (2 g/kg body weight), and blood glucose was measured immediately before and at 15, 30, 45, 60, and 120 min post i.p. injection.

For insulin assays, tail vein blood was collected from ad libitum chow-fed mice. Serum was collected by centrifugation and assayed by a commercially available ELISA kit (Crystal Chem. Inc., Downers Grove, IL) with sensitivity from 100–6400 pg/ml and intra- and inter-assay coefficient of variance of less than 10%. Only data that satisfied the manufacturer's performance characteristics were used.

### Statistics

The data are reported as mean ± SEM. All statistical analyses were performed using Prism (version 5.0) software. ITT and GTTs were analyzed by comparing the mean of the Area Under the Curves by t-test. Groups of more than two and individual weight-gain time points were analyzed by a Bonferroni's post-hoc test following a one-way ANOVA. When planned comparisons had been part of the experimental design, a Bonferroni post-hoc analysis was used to assess selected pairs of means. T-tests were used to compare results between groups of two. Fisher's exact test was used to analyze differences in cyclicity percentages. All statistical comparisons were made between data sets with equal variances. P<0.05 was considered statistically significant.

## Results

**Figure 1:**
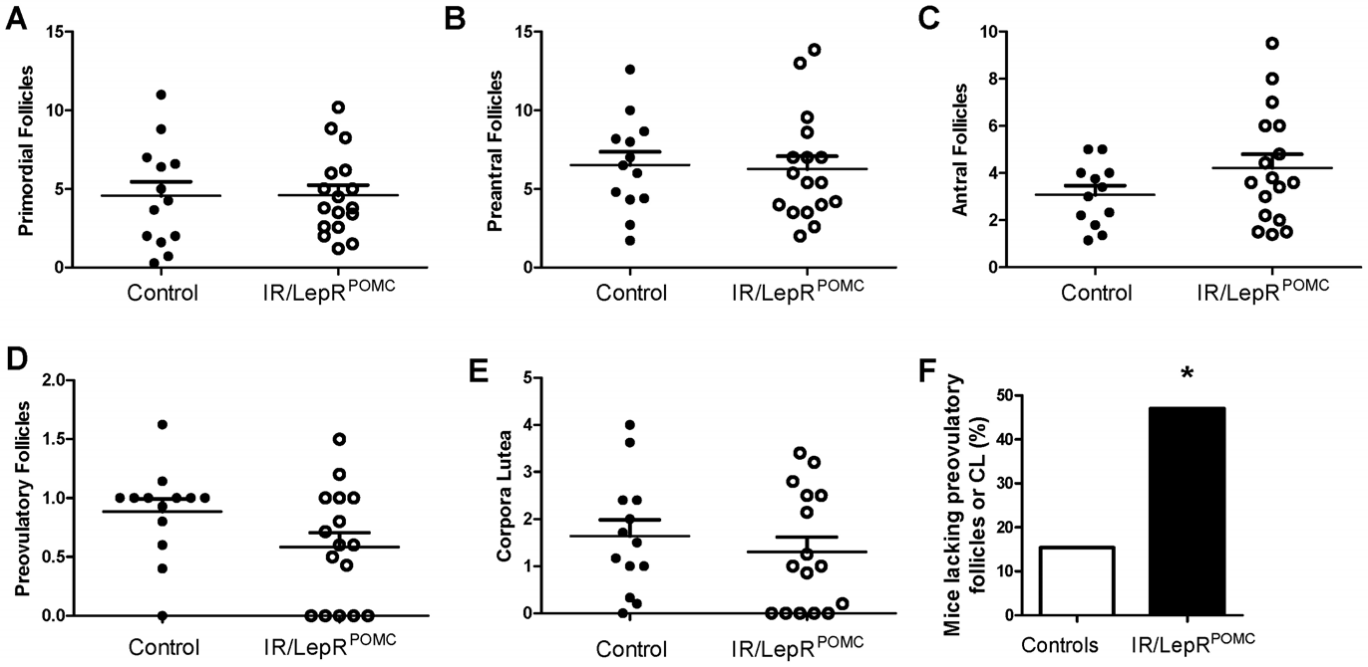
Ovarian Morphology of control and IR/LepR^POMC^ females. A-E. Ovarian sections throughout the ovaries of individual control and IR/LepR^POMC^ females were examined for follicle types shown, n = 13–17. F. Ovarian sections throughout the ovaries of control and IR/LepR^POMC^ females were examined and the percentage of ovaries lacking either preovulatory follicles or corpora lutea (or both) is shown, n = 13–17. Statistical significance was calculated using Fisher's exact test (p = 0.0481).

To evaluate the reduced fertility of IR/LepR^POMC^ mice [37], we examined their ovarian morphology. All follicle stages were found (Fig. 1); however, approximately 45% of IR/LepR^POMC^ mice lacked either corpora lutea or preovulatory follicles (Fig. 1 d,3,f). In particular, 5 of 16 IR/LepR^POMC^ females showed no ovarian corpora lutea, while only 1 of 13 females showed no corpora lutea. The data show histological evidence compatible with anovulation in the form of a lack of preovulatory follicles and corpora lutea. These findings are consistent with impaired late-stage follicle maturation and ovulation.

**Figure 2:**
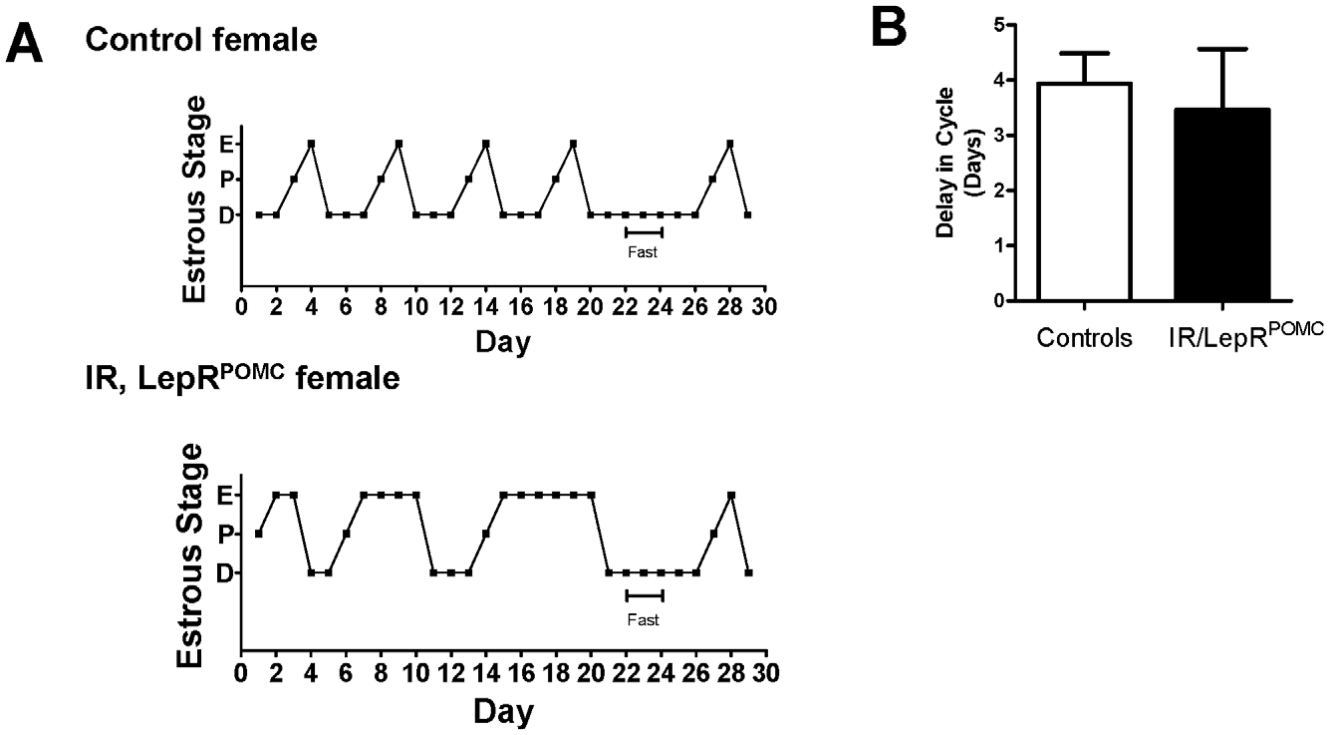
IR/LepR^POMC^ females have reduced ovulation but show normal cycling suppression from fasting. A. Example of delay in resumption of estrous cycles measured by vaginal cytology taken before, during, and after a 48 hour fast. B. Delay from fast quantified for control (white bars) or IR/LepR^POMC^ (black bars) mice, n = 9–13 (not significant). Mean ± SEM.

We then tested whether impaired ovulation in IR/LepR^POMC^ mice results from POMC neurons failing to communicate key permissive signals of energy sufficiency to GnRH neurons. Contrary to expectations, fasting induced a similar lengthening of estrous cycles in both IR/LepR^POMC^ females and control littermates (Fig. 2a,b).

**Figure 3:**
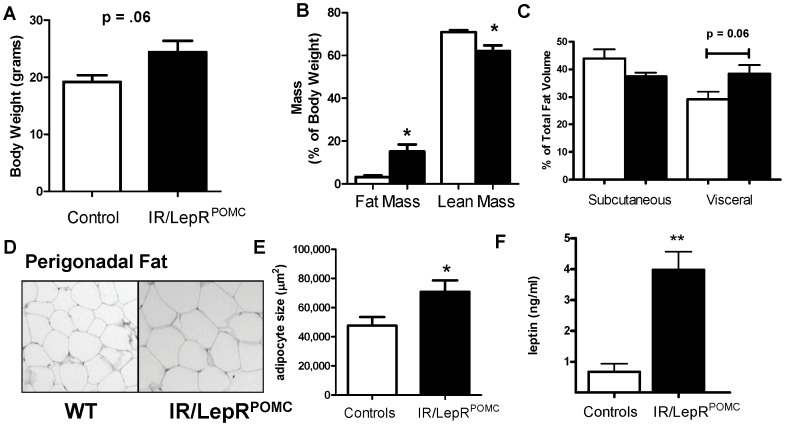
Increased fat mass and intra-abdominal adipocyte hypertrophy in IR/LepR^POMC^ mice. A. Body weight of IR/LepR^POMC^ female mice and controls. B. Fat and lean mass of IR/LepR^POMC^ females and controls measured by NMR (white bars  =  control mice, black bars  =  IR/LepR^POMC^ mice), n = 5–6. C. Subcutaneous and intra-abdominal fat volume measured by CT scan in IR/LepR^POMC^ females and controls, n = 5–6 D. Section of perigonadal fat tissue, paraffin embedded and stained with H/E. Each panels are shown at 200x. E. Adipocyte size calculated from H/E stained perigonadal fat pads, 50 cells counted per section/mouse, n = 15–24 mice. * indicates p<0.05. F. Serum leptin levels of control (white bars) or IR/LepR^POMC^ (black bars) mice 3 hours after chow removal. n = 4, ** indicates p<0.01. Mean ± SEM.

At 6 months of age, IR/LepR^POMC^ mice on a mixed-strain background show increased body weight compared with wildtype controls because of fat deposition [37]. To further clarify the metabolic phenotype of these mice, we examined whether the body composition of IR/LepR^POMC^ females changes before becoming overtly overweight. We confirmed that at 4 months old, absolute body weight was not statistically increased in these mice, although a trend was seen (Fig. 3a). However, the percent fat mass was elevated and percent lean mass correspondingly decreased (Fig. 3b). Lean mass did not decrease in absolute terms (not shown). This finding was reflected by a trend toward increased intra-abdominal adiposity in IR/LepR^POMC^ females and the presence of perigonadal adipocyte hypertrophy (Fig. 3c-e). Correspondingly, circulating levels of the adipokine leptin were also elevated in IR/LepR^POMC^ females (Fig. 3f).

**Figure 4:**
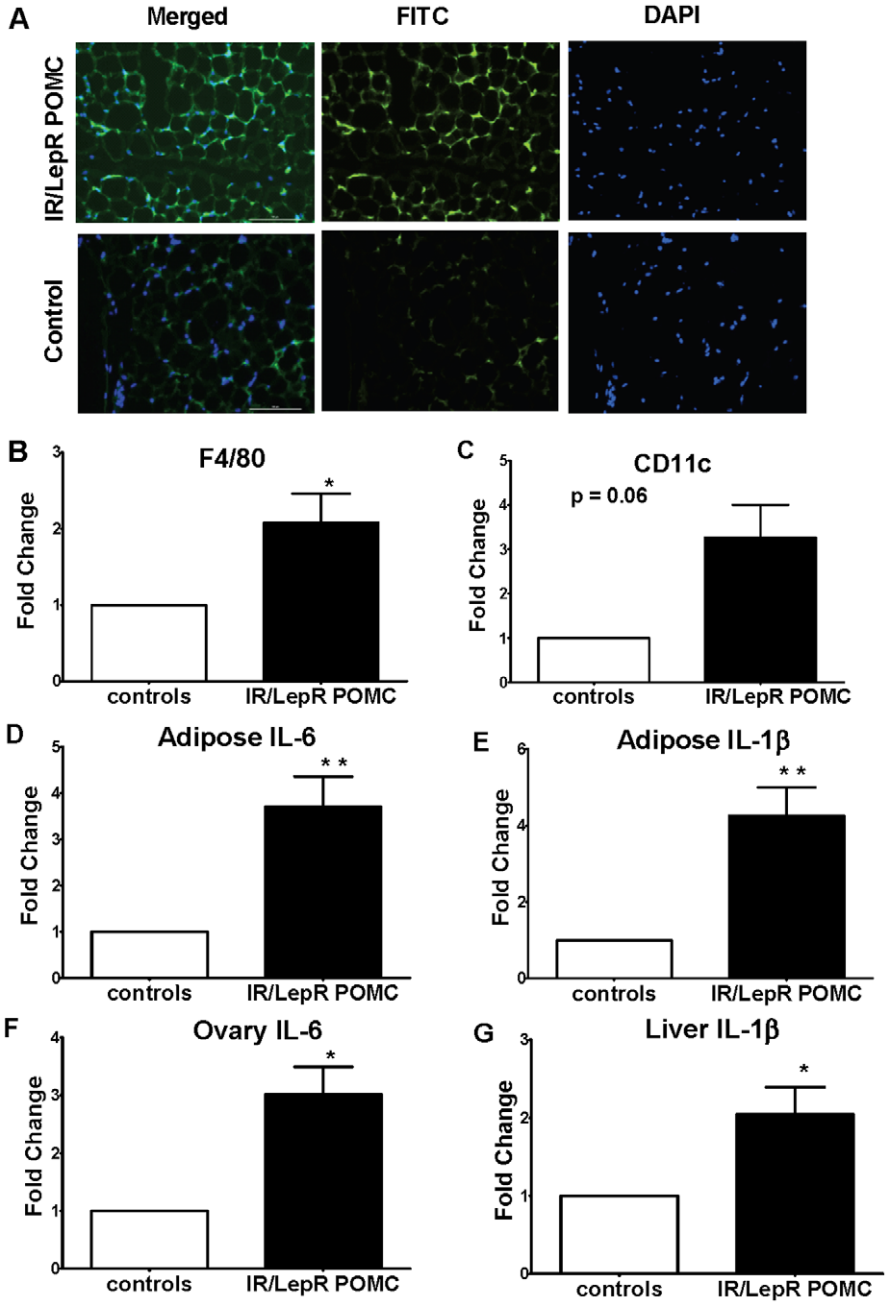
IR/LepR^POMC^ Females have tissue-specific low-grade inflammation. A. Immunostaining for macrophage marker F4/80 in adipose tissue. F4/80 staining (FITC) was merged with DAPI to identify activated macrophages. B. Adipose tissue F4/80 gene expression. C. Adipose tissue CD11c gene expression. D. Adipose tissue IL-6 gene expression. E. Adipose tissue IL-1β gene expression. F. Ovary IL-6 gene expression. G. Liver IL-1β gene expression. Mean ± SEM, n = 4–5 *  =  p<.05 and **  =  p<.01 compared with controls.

We next examined whether hypertrophy in the perigonadal fat depot was associated with deleterious inflammatory cell accumulation and cytokine production. We found elevated macrophage accumulation as shown by significantly increased F4/80 staining in the perigonadal fat of IR/LepR^POMC^ mice compared with IR/LepR controls (Fig. 4a,b). Gene expression of CD11c, an antigen typically expressed by pro-inflammatory macrophages, was approximately 3-fold elevated compared with controls (Fig. 4c). Additionally, gene transcript levels of inflammatory cytokines IL-6 and IL-1β were increased (Fig. 4d,e), suggesting that the macrophage infiltration was associated with increased low-grade inflammation in this tissue. No difference in adipose tissue TNF-α or IL-10 was found (data not shown).

Because hepatic insulin resistance is associated with local inflammation [19], [43], [44], we also examined cytokine expression in the liver. Liver IL-1β mRNA levels were also elevated, suggesting an inflammatory state in hepatic tissue as well (Fig. 4g). Finally, because any disruption of the normal inflammation that accompanies ovulation [45] could potentially disrupt ovarian function, we compared inflammatory markers in the ovaries of control and targeted mutant mice. Ovarian IL-6 mRNA levels were increased on diestrus (Fig 4f). Gene transcript levels for ovarian F4/80, CD11c, TNFalpha, and IL-1β were not changed compared with control mice (not shown). Because cytokines can stimulate proliferation of theca cells [82], we used a marker of cell proliferation to examine this parameter in the ovaries of these mice. In IR/LepR^POMC^ mice, PCNA immunostaining showed no difference in theca cell proliferation compared to controls (data not shown). Finally, no differences were found in circulating levels of cytokines or CRP (Table 1).

**Table 1:** Serum inflammatory markers are normal in IR/LepR^POMC^ Females.

Cytokine	Control	IR/LepR^POMC^
IL-1β (pg/ml)	890.0±188.9	772.2±80.22
Il-4 (pg/ml)	12.87±1.66	13.52±1.57
IL-6 (pg/ml)	19.62±4.34	18.48±1.96
IL-10 (pg/ml)	190.7±57.19	279.0±33.31
TNF-α (pg/ml)	591.4±85.86	631.5±86.37
IL-13 (pg/ml)	2364.0±240.8	3578.0±493.0
INF-γ (pg/ml)	471.7±80.79	556.1±80.62
MCP-1 (pg/ml)	462.1±68.64	445.2±57.47
CRP (mg/ml)	3.04±0.17	2.89±.28

Cytokine levels were measured in serum by Bio-Plex cytokine array, n = 4–5. Mean ± SEM.

Tumor Necrosis Factor-alpha (TNF-α), Interferon-gamma (INF-γ),

monocyte chemotactic protein-1 (MCP-1).

**Figure 5:**
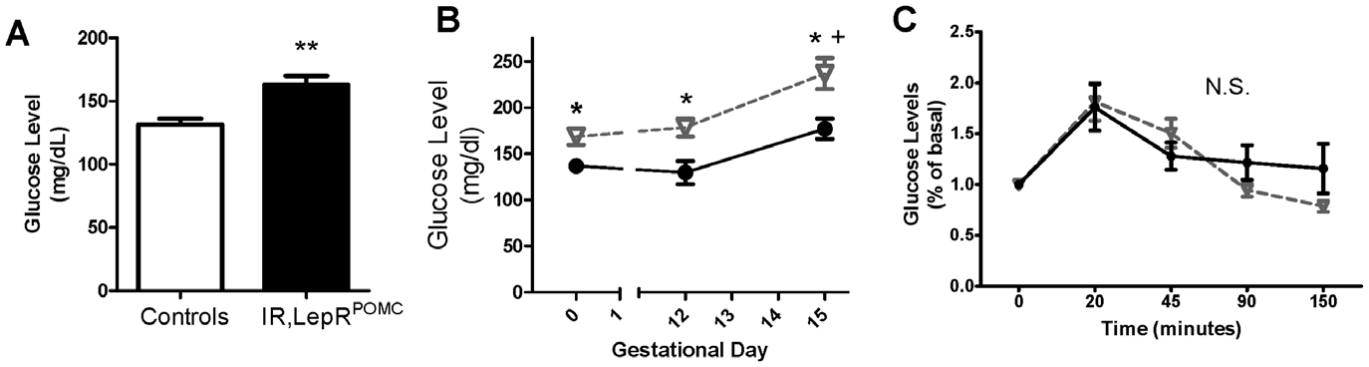
IR/LepR^POMC^ females are hyperglycemic and show hyperglycemia during pregnancy. A. IR/LepR^POMC^ female glucose levels under basal conditions (n = 6–7) ** p<0.01. B. Fasted glucose levels in a second cohort of females, before pregnancy and on gestational day 12 and 15 (Black circles are control dams, open triangles are IR/LepR^POMC^ dams; n = 11–12) * p<0.05, compared with controls at same timepoint; ^+^ p<0.05, compared with pre-pregnancy values of same group. C. Glucose tolerance testing (2 g/kg) performed on day 15–18 of gestation. (Black circles are control dams, open triangles are IR/LepR^POMC^ dams; n = 6). Mean ± SEM.

IR/LepR^POMC^ females were reported to have impaired glucose tolerance and elevated basal fasting blood glucose levels at 2 months old before beginning a hyperglycemic euglycemic clamp [46]. Indeed, we confirmed that blood glucose levels are elevated in these mice at 3 months of age (Fig. 5a). To resolve whether glucose regulation in IR/LepR^POMC^ females is abnormal during pregnancy, we examined fasting blood glucose and insulin levels at day 12 and 15 of gestation and performed a glucose tolerance tests between gestational days 15–18. We found a significant elevation in fasting glucose levels at gestational days 12 and 15 in IR/LepR^POMC^ females (Fig. 5b), but insulin levels failed to rise in compensation (0.481 pg/ml +/− 0.0439 controls vs. 0.654 ng/ml +/− 0.294 experimental mice, n = 5). Glucose tolerance was not impaired when normalized to initial glucose levels (Fig 5c), indicating that pregnancy induced a similar increase in blood glucose levels in each group of mice. In addition, HOMA-IR values did not differ between the two groups (not shown). Therefore, IR/LepR^POMC^ mice are hyperglycemic before and during pregnancy, without evidence of gestational glucose intolerance.

## Discussion

Mice lacking leptin receptors and insulin receptors in POMC cells (IR/LepR^POMC^ mice) were originally developed to study the control of food consumption by POMC neurons in the hypothalamus [37]. Both insulin and the adipokine leptin communicate the status of adiposity stores to the brain [47], [48]. POMC neurons modulate food intake, energy use, and hepatic glucose production, and may change these parameters independently [34], [35], [36]. High parasympathetic output (or low sympathetic output) transmitted through the vagus nerve to the liver reduces hepatic glucose production by inhibiting hepatic enzymes involved in gluconeogenesis and by activating enzymes promoting glycogen synthesis. This output is modulated by neurons of the hypothalamus, including POMC neurons [49], [50] and seems to result in excess HGP in IR/LepR^POMC^ mice. Likewise, POMC neurons project multisynaptically to brown adipose tissue [51] and can thereby change oxygen consumption and basal metabolic rate. Given the suppressed oxygen consumption in IR/LepR^POMC^ mice without changed physical activity levels [37], this pathway likely underlies the increased adiposity in older IR/LepR^POMC^ mice.

Both leptin and insulin signaling in POMC neurons can change neuronal activity as well as the production and processing of the POMC gene product [37], [52]. Importantly, a single nucleotide polymorphism of the POMC gene is associated with PCOS risk in women [53]. Hence, further characterization of POMC neuronal function and regulation is important to understand the potential contribution of this pathway to PCOS.

We have recently shown that the IR/LepR^POMC^ mouse displays hyperandrogenemia, lengthened estrous cycles, and reduced fertility, arguably qualifying it for a PCOS “diagnosis.” However, the ovaries of IR/LepR^POMC^ mice do not show excess numbers of small antral follicles, i.e. polycystic ovaries. Most women with polycystic ovaries are asymptomatic [54], and diagnosis of PCOS does not require the presence of polycystic ovaries [55]. Thus, the IR/LepR^POMC^ mouse might best serve a model of the subset of PCOS patients diagnosed because of impaired ovulation and hyperandrogenemia. Interestingly, not all IR/LepR^POMC^ mice are affected by anovulation or infertility. The cause of the incomplete penetrance of this phenotype is the subject of ongoing studies.

The primary cause of reproductive impairment in IR/LepR^POMC^ mice has not yet been resolved. One possible explanation for the impaired fertility in IR/LepR^POMC^ mice is that POMC neurons fail to communicate key permissive signals of energy sufficiency to GnRH neurons because of their lack of insulin and leptin sensing. If GnRH neurons were to perceive a constant state of energy deprivation, the HPG axis should be chronically suppressed. However, fasting induced a similar lengthening of estrous cycles in both IR/LepR^POMC^ females and control littermates. With our previous findings of robust LH secretion in these mice [37], it seems likely that the requirement for brain leptin and insulin signaling to maintain GnRH output [56], [57] depends on signaling through other neuronal types.

These reproductive traits are accompanied by elevated circulating LH and insulin, reduced insulin sensitivity, and normal FSH, prolactin, and estradiol levels [58]. Here, we have confirmed and extended our findings of impaired ovulation and glucose handling. Furthermore, our data show that IR/LepR^POMC^ mice develop adipocyte hypertrophy and tissue-specific inflammation. Thus, this study presents the first evidence for adipose tissue dysfunction in this model of PCOS.

Characterizing adipose tissue defects in an animal model of PCOS is clinically relevant because adipocyte dysfunction could be the earliest event in the development of metabolic or cardiovascular complications in the PCOS population. Although obesity contributes to the severity of PCOS in many women [2], [59], [60], controversy exists about the impact of depot location and other factors. Abdominal adiposity has been reported to be common [6], [61] and to increase preferentially with weight gain in PCOS patients [62], worsening their metabolic and reproductive abnormalities [63], [64]. Loss of intra-abdominal fat may be associated with resumption of ovulation [65]. Nevertheless, findings of increased intra-abdominal fat in women with PCOS have been inconsistent [66], [67], [68]. Alternatively, the expandability of the number and size of adipocytes may be exhausted in women with PCOS. Among women with PCOS, adipocyte diameter was recently reported to be increased by 25% compared with that in BMI-matched control women who do not have PCOS [5]. These effects may be driven by hyperandrogenemia; prenatal androgen exposure results in increased visceral adipocyte size, with insulin resistance and glucose intolerance [69]. We have shown that abdominal adipocyte hypertrophy occurs in IR/LepR^POMC^ mice. Normal CNS leptin signaling promotes energy use, thus reducing energy storage in fat tissue [70]. For its part, brain insulin signaling promotes lipogenesis and increased lipoprotein lipase expression in adipose tissue [71]. Thus, one explanation for the PCOS-like metabolic phenotype of the IR/LepR^POMC^ mice (remaining to be tested) is that inadequate adipogenesis in the face of a positive energy balance drives adipocyte dysfunction, chronic inflammation, and insulin resistance.

Although circulating markers of chronic inflammation are elevated in women with PCOS, the source of their production is unclear. Manneras-Holm and colleagues found no increase in macrophage density in subcutaneous adipose tissue from women with PCOS [5]. The antibody used in that study, however, may also detect preadipocytes [72], [73], [74], [75], [76], whose number would be expected to be reduced in hyperinsulinemic patients [77]. In addition, a positive control showing an association between obesity and macrophage density was not included in the study design. Thus, the question of whether inflammation occurs in the adipose tissue of PCOS patients or elsewhere is unresolved. We have shown tissue-specific rather than a general elevation of circulating cytokines in IR/LepR^POMC^ mice. It remains to be established whether a more generalized inflammatory state occurs later in the life of these animals. However, our results suggest that investigation of the inflammatory state of adipose and other tissues in people with PCOS should continue. In addition, our data argue against circulating inflammatory signals serving as a causal factor in PCOS-like metabolic impairments, at least in this model.

Chronic inflammation may contribute to the hyperandrogenemia of PCOS [78]. In vitro studies have shown the ability of pro-inflammatory stimuli to upregulate the steroidogenic enzyme responsible for androgen production in ovarian theca cells [79], [80], [81]. Cytokines can also stimulate proliferation of theca cells [82]. In addition, abnormal inflammatory input may impair the finely tuned inflammatory processes used to control ovulation [45], [83], [84]. In that light, the excess cytokine gene expression in the ovary of IR/LepR^POMC^ mice is intriguing. Given that we saw no evidence of theca cell proliferation, additional studies will be required to identify the cause and consequence of increased ovarian IL-6 mRNA in these mice.

The progression from insulin resistance to type 2 diabetes mellitus (T2D) can take years, although women with PCOS might show a more rapid conversion from impaired glucose tolerance to T2D [85], [86]. Gestational diabetes (GDM) is an early marker of T2D risk and may constitute an early stage in the progression to that disease [87]. Indeed, women with PCOS are at increased risk for developing GDM [88]. Gestational diabetes results from impaired adaptation of the pancreatic beta cells during pregnancy [89]. If adipocyte dysfunction and the other PCOS-like traits of IR/LepR^POMC^ mice increases T2D risk, we hypothesized these animals would show impaired glucose tolerance during pregnancy. Current and proposed clinical guidelines allow diagnosis of GDM based on either elevated fasting glucose levels or abnormal oral GTT results [90], [91]. IR/LepR^POMC^ mice continued to show elevated fasting glucose levels during pregnancy that rose significantly by d15 without an accompanying rise in insulin levels. However, glucose tolerance, once normalized for starting glucose levels, was no worse than control mice. No absolute values for hyperglycemia during GTTs have been established for mice, making comparison to the situation in the clinic difficult. Nevertheless, IR/LepR^POMC^ mice appear to have preexisting hyperglycemia worsened by pregnancy. It remains to be seen whether high fat diet would further stress their glucose regulatory systems and reveal frank T2D.

## Conclusion

In summary, the results of this study show tissue-specific inflammation in IR/LepR^POMC^ mice, a new mouse model for PCOS, as shown by macrophage infiltration in adipose tissue, and elevated cytokine mRNA levels in adipose tissue, liver, and ovary. Structural changes also occurred in adipose tissue, as shown by perigonadal adipocyte hypertrophy. These mice also showed susceptibility to gestational hyperglycemia. These findings are consistent with the characteristics of PCOS patients that may contribute to the insulin resistance and type 2 diabetes risk associated with this common disorder. Future studies will be directed at resolving whether the adipocyte dysfunction is directly induced by hyperandrogenemia or by secondary metabolic alterations such as obesity.

## References

[1] Azziz R, Woods KS, Reyna R, Key TJ, Knochenhauer ES, et al (2004) The prevalence and features of the polycystic ovary syndrome in an unselected population. J Clin Endocrinol Metab 89: 2745–2749. 15181052 10.1210/jc.2003-032046

[2] Moran LJ, Misso ML, Wild RA, Norman RJ (2010) Impaired glucose tolerance, type 2 diabetes and metabolic syndrome in polycystic ovary syndrome: a systematic review and meta-analysis. Hum Reprod Update 16: 347–363. 20159883 10.1093/humupd/dmq001

[3] Lo JC, Feigenbaum SL, Yang J, Pressman AR, Selby JV, et al (2006) Epidemiology and adverse cardiovascular risk profile of diagnosed polycystic ovary syndrome. J Clin Endocrinol Metab 91: 1357–1363. 16434451 10.1210/jc.2005-2430

[4] Christian RC, Dumesic DA, Behrenbeck T, Oberg AL, Sheedy PF 2nd, et al (2003) Prevalence and predictors of coronary artery calcification in women with polycystic ovary syndrome. J Clin Endocrinol Metab 88: 2562–2568. 12788855 10.1210/jc.2003-030334

[5] Manneras-Holm L, Leonhardt H, Kullberg J, Jennische E, Oden A, et al (2011) Adipose tissue has aberrant morphology and function in PCOS: enlarged adipocytes and low serum adiponectin, but not circulating sex steroids, are strongly associated with insulin resistance. J Clin Endocrinol Metab 96: E304–311. 21084397 10.1210/jc.2010-1290

[6] Carmina E, Bucchieri S, Esposito A, Del Puente A, Mansueto P, et al (2007) Abdominal fat quantity and distribution in women with polycystic ovary syndrome and extent of its relation to insulin resistance. J Clin Endocrinol Metab 92: 2500–2505. 17405838 10.1210/jc.2006-2725

[7] Kuchenbecker WKH, Groen H, van Asselt SJ, Bolster JHT, Zwerver J, et al (2011) In women with polycystic ovary syndrome and obesity, loss of intra-abdominal fat is associated with resumption of ovulation. Human Reproduction 26: 2505–2512. 21771766 10.1093/humrep/der229

[8] Karabulut A, Yaylali GF, Demirlenk S, Sevket O, Acun A (2012) Evaluation of body fat distribution in PCOS and its association with carotid atherosclerosis and insulin resistance. Gynecological Endocrinology 28: 111–114. 21770828 10.3109/09513590.2011.589929

[9] Weyer C, Foley JE, Bogardus C, Tataranni PA, Pratley RE (2000) Enlarged subcutaneous abdominal adipocyte size, but not obesity itself, predicts Type II diabetes independent of insulin resistance. Diabetologia 43: 1498–1506. 11151758 10.1007/s001250051560

[10] Joe AWB, Yi L, Even Y, Vogl AW, Rossi FMV (2009) Depot-Specific Differences in Adipogenic Progenitor Abundance and Proliferative Response to High-Fat Diet. Stem Cells 27: 2563–2570. 19658193 10.1002/stem.190

[11] Kershaw EE, Flier JS (2004) Adipose tissue as an endocrine organ. J Clin Endocrinol Metab 89: 2548–2556. 15181022 10.1210/jc.2004-0395

[12] Fontana L, Eagon JC, Trujillo ME, Scherer PE, Klein S (2007) Visceral fat adipokine secretion is associated with systemic inflammation in obese humans. Diabetes 56: 1010–1013. 17287468 10.2337/db06-1656

[13] Sun K, Kusminski CM, Scherer PE (2011) Adipose tissue remodeling and obesity. J Clin Invest 121: 2094–2101. 21633177 10.1172/JCI45887 PMC3104761

[14] Halberg N, Khan T, Trujillo ME, Wernstedt-Asterholm I, Attie AD, et al (2009) Hypoxia-inducible factor 1alpha induces fibrosis and insulin resistance in white adipose tissue. Mol Cell Biol 29: 4467–4483. 19546236 10.1128/MCB.00192-09 PMC2725728

[15] Weisberg SP, McCann D, Desai M, Rosenbaum M, Leibel RL, et al (2003) Obesity is associated with macrophage accumulation in adipose tissue. J Clin Invest 112: 1796–1808. 14679176 10.1172/JCI19246 PMC296995

[16] Xu H, Barnes GT, Yang Q, Tan G, Yang D, et al (2003) Chronic inflammation in fat plays a crucial role in the development of obesity-related insulin resistance. J Clin Invest 112: 1821–1830. 14679177 10.1172/JCI19451 PMC296998

[17] Zhang HM, Chen LL, Wang L, Xu S, Wang X, et al (2009) Macrophage infiltrates with high levels of Toll-like receptor 4 expression in white adipose tissues of male Chinese. Nutr Metab Cardiovasc Dis 19: 736–743. 19356913 10.1016/j.numecd.2008.12.016

[18] Chen Z, Torrens JI, Anand A, Spiegelman BM, Friedman JM (2005) Krox20 stimulates adipogenesis via C/EBPbeta-dependent and -independent mechanisms. Cell Metab 1: 93–106. 16054051 10.1016/j.cmet.2004.12.009

[19] Cai D, Yuan M, Frantz DF, Melendez PA, Hansen L, et al (2005) Local and systemic insulin resistance resulting from hepatic activation of IKK-beta and NF-kappaB. Nat Med 11: 183–190. 15685173 10.1038/nm1166 PMC1440292

[20] Austin RL, Rune A, Bouzakri K, Zierath JR, Krook A (2008) SiRNA-mediated reduction of inhibitor of nuclear factor-kappa B kinase prevents tumor necrosis factor-alpha-induced insulin resistance in human skeletal muscle. Diabetes 57: 2066–2073. 18443205 10.2337/db07-0763 PMC2494681

[21] Shoelson SE, Lee J, Goldfine AB (2006) Inflammation and insulin resistance. J Clin Invest 116: 1793–1801. 16823477 10.1172/JCI29069 PMC1483173

[22] Goldfine AB, Silver R, Aldhahi W, Cai D, Tatro E, et al (2008) Use of salsalate to target inflammation in the treatment of insulin resistance and type 2 diabetes. Clin Transl Sci 1: 36–43. 19337387 10.1111/j.1752-8062.2008.00026.x PMC2662587

[23] Xu HY, Barnes GT, Yang Q, Tan Q, Yang DS, et al (2003) Chronic inflammation in fat plays a crucial role in the development of obesity-related insulin resistance. Journal of Clinical Investigation 112: 1821–1830. 14679177 10.1172/JCI19451 PMC296998

[24] Cai DS, Yuan MS, Frantz DF, Melendez PA, Hansen L, et al (2005) Local and systemic insulin resistance resulting from hepatic activation of IKK-beta and NF-kappa B. Nature Medicine. 11: 183–190. 10.1038/nm1166 PMC1440292 15685173

[25] Sprague AH, Khalil RA (2009) Inflammatory cytokines in vascular dysfunction and vascular disease. Biochem Pharmacol 78: 539–552. 19413999 10.1016/j.bcp.2009.04.029 PMC2730638

[26] Chen MJ, Chen HF, Chen SU, Ho HN, Yang YS, et al (2009) The relationship between follistatin and chronic low-grade inflammation in women with polycystic ovary syndrome. Fertility and Sterility 92: 2041–2044. 19591997 10.1016/j.fertnstert.2009.06.009

[27] Gonzalez F, Rote NS, Minium J, Kirwan JP (2006) Increased activation of nuclear factor kappaB triggers inflammation and insulin resistance in polycystic ovary syndrome. J Clin Endocrinol Metab 91: 1508–1512. 16464947 10.1210/jc.2005-2327

[28] Tarkun I, Cetinarslan B, Turemen E, Canturk Z, Biyikli M (2006) Association between Circulating Tumor Necrosis Factor-Alpha, Interleukin-6, and Insulin Resistance in Normal-Weight Women with Polycystic Ovary Syndrome. Metab Syndr Relat Disord 4: 122–128. 18370758 10.1089/met.2006.4.122

[29] Gonzalez F, Rote NS, Minium J, Weaver AL, Kirwan JP (2010) Elevated circulating levels of macrophage migration inhibitory factor in polycystic ovary syndrome. Cytokine 51: 240–244. 20598902 10.1016/j.cyto.2010.06.008 PMC2914837

[30] Kelly CC, Lyall H, Petrie JR, Gould GW, Connell JM, et al (2001) Low grade chronic inflammation in women with polycystic ovarian syndrome. J Clin Endocrinol Metab 86: 2453–2455. 11397838 10.1210/jcem.86.6.7580

[31] Puder JJ, Varga S, Kraenzlin M, De Geyter C, Keller U, et al (2005) Central fat excess in polycystic ovary syndrome: relation to low-grade inflammation and insulin resistance. J Clin Endocrinol Metab 90: 6014–6021. 16105965 10.1210/jc.2005-1002

[32] Franks S (2009) Do Animal Models of Polycystic Ovary Syndrome Help to Understand Its Pathogenesis and Management? Yes, but Their Limitations Should be Recognized. Endocrinology 150: 3983–3985. 19700609 10.1210/en.2009-0652

[33] Szukiewicz D, Uilenbroek JTJ (1998) Polycystic ovary syndrome - Searching for an animal model. Journal of Medicine 29: 259–275. 10503163

[34] De Jonghe BC, Hayes MR, Banno R, Skibicka KP, Zimmer DJ, et al (2011) Deficiency of PTP1B in POMC neurons leads to alterations in energy balance and homeostatic response to cold exposure. American Journal of Physiology-Endocrinology and Metabolism 300: E1002–E1011. 21406615 10.1152/ajpendo.00639.2010 PMC3118594

[35] Ramadori G, Fujikawa T, Fukuda M, Anderson J, Morgan DA, et al (2010) SIRT1 Deacetylase in POMC Neurons Is Required for Homeostatic Defenses against Diet-Induced Obesity. Cell Metabolism 12: 78–87. 20620997 10.1016/j.cmet.2010.05.010 PMC2904327

[36] Lin HV, Plum L, Ono H, Gutierrez-Juarez R, Shanabrough M, et al (2010) Divergent Regulation of Energy Expenditure and Hepatic Glucose Production by Insulin Receptor in Agouti-Related Protein and POMC Neurons. Diabetes 59: 337–346. 19933998 10.2337/db09-1303 PMC2809966

[37] Hill JW, Elias CF, Fukuda M, Williams KW, Berglund ED, et al (2010) Direct insulin and leptin action on pro-opiomelanocortin neurons is required for normal glucose homeostasis and fertility. Cell Metab 11: 286–297. 20374961 10.1016/j.cmet.2010.03.002 PMC2854520

[38] Carmina E, Lobo RA (2004) Use of fasting blood to assess the prevalence of insulin resistance in women with polycystic ovary syndrome. Fertility and Sterility 82: 661–665. 15374711 10.1016/j.fertnstert.2004.01.041

[39] DeUgarte CM, Bartolucci AA, Azziz R (2005) Prevalence of insulin resistance in the polycystic ovary syndrome using the homeostasis model assessment. Fertility and Sterility 83: 1454–1460. 15866584 10.1016/j.fertnstert.2004.11.070

[40] Yildiz BO, Knochenhauer ES, Azziz R (2008) Impact of obesity on the risk for polycystic ovary syndrome. Journal of Clinical Endocrinology & Metabolism 93: 162–168. 17925334 10.1210/jc.2007-1834 PMC2190739

[41] Villa J, Pratley RE (2011) Adipose Tissue Dysfunction in Polycystic Ovary Syndrome. Current Diabetes Reports 11: 179–184. 21424395 10.1007/s11892-011-0189-8

[42] Bingel AS, Schwartz NB (1969) Pituitary LH content and reproductive tract changes during the mouse oestrous cycle. J Reprod Fertil 19: 215–222. 5815724 10.1530/jrf.0.0190215

[43] Zhan YT, An W (2010) Roles of liver innate immune cells in nonalcoholic fatty liver disease. World J Gastroenterol 16: 4652–4660. 20872965 10.3748/wjg.v16.i37.4652 PMC2951515

[44] Li Z, Diehl AM (2003) Innate immunity in the liver. Curr Opin Gastroenterol 19: 565–571. 15703606 10.1097/00001574-200311000-00009

[45] Oakley OR, Kim H, El-Amouri I, Lin PC, Cho J, et al (2010) Periovulatory leukocyte infiltration in the rat ovary. Endocrinology 151: 4551–4559. 20591976 10.1210/en.2009-1444 PMC2940505

[46] Hill JW, Elias CF, Fukuda M, Williams KW, Berglund ED, et al. Direct insulin and leptin action on pro-opiomelanocortin neurons is required for normal glucose homeostasis and fertility. Cell Metab 11: 286–297. 20374961 10.1016/j.cmet.2010.03.002 PMC2854520

[47] Benoit SC, Air EL, Coolen LM, Strauss R, Jackman A, et al (2002) The catabolic action of insulin in the brain is mediated by melanocortins. J Neurosci 22: 9048–9052. 12388611 10.1523/JNEUROSCI.22-20-09048.2002 PMC6757684

[48] Elmquist JK, Bjorbaek C, Ahima RS, Flier JS, Saper CB (1998) Distributions of leptin receptor mRNA isoforms in the rat brain. J Comp Neurol 395: 535–547. 9619505

[49] Marino JS, Xu Y, Hill JW (2011) Central insulin and leptin-mediated autonomic control of glucose homeostasis. Trends Endocrinol Metab 22: 275–285. 21489811 10.1016/j.tem.2011.03.001 PMC5154334

[50] Kalsbeek A, Bruinstroop E, Yi CX, Klieverik LP, La Fleur SE, et al (2010) Hypothalamic control of energy metabolism via the autonomic nervous system. Ann N Y Acad Sci 1212: 114–129. 21070249 10.1111/j.1749-6632.2010.05800.x

[51] Oldfield BJ, Giles ME, Watson A, Anderson C, Colvill LM, et al (2002) The neurochemical characterisation of hypothalamic pathways projecting polysynaptically to brown adipose tissue in the rat. Neuroscience 110: 515–526. 11906790 10.1016/s0306-4522(01)00555-3

[52] Wardlaw SL (2011) Hypothalamic proopiomelanocortin processing and the regulation of energy balance. Eur J Pharmacol 660: 213–219. 21208604 10.1016/j.ejphar.2010.10.107 PMC3095770

[53] Ewens KG, Stewart DR, Ankener W, Urbanek M, McAllister JM, et al (2010) Family-based analysis of candidate genes for polycystic ovary syndrome. J Clin Endocrinol Metab 95: 2306–2315. 20200332 10.1210/jc.2009-2703 PMC2869537

[54] Kristensen SL, Ramlau-Hansen CH, Ernst E, Olsen SF, Bonde JP, et al (2010) A very large proportion of young Danish women have polycystic ovaries: is a revision of the Rotterdam criteria needed? Human Reproduction 25: 3117–3122. 20940139 10.1093/humrep/deq273

[55] Goodarzi MO, Dumesic DA, Chazenbalk G, Azziz R (2011) Polycystic ovary syndrome: etiology, pathogenesis and diagnosis. Nature Reviews Endocrinology 7: 219–231. 10.1038/nrendo.2010.217 21263450

[56] Bruning JC, Gautam D, Burks DJ, Gillette J, Schubert M, et al (2000) Role of brain insulin receptor in control of body weight and reproduction. Science 289: 2122–2125. 11000114 10.1126/science.289.5487.2122

[57] de Luca C, Kowalski TJ, Zhang Y, Elmquist JK, Lee C, et al (2005) Complete rescue of obesity, diabetes, and infertility in db/db mice by neuron-specific LEPR-B transgenes. J Clin Invest 115: 3484–3493. 16284652 10.1172/JCI24059 PMC1280964

[58] Hill JW, Elias CF, Fukuda M, Williams KW, Berglund ED, et al (2010) Direct Insulin and Leptin Action on Pro-opiomelanocortin Neurons Is Required for Normal Glucose Homeostasis and Fertility. Cell Metabolism 11: 286–297. 20374961 10.1016/j.cmet.2010.03.002 PMC2854520

[59] Marshall JC, Hart AD (2006) Editorial: Obesity in adolescent girls: Is excess androgen the real bad actor? Journal of Clinical Endocrinology & Metabolism 91: 393–395. 16461952 10.1210/jc.2005-2665

[60] Pasquali R, Gambineri A, Pagotto U (2006) The impact of obesity on reproduction in women with polycystic ovary syndrome. BJOG 113: 1148–1159. 16827825 10.1111/j.1471-0528.2006.00990.x

[61] Battaglia C, Battaglia B, Mancini F, Paradisi R, Fabbri R, et al (2011) Ultrasonographic extended-view technique for evaluation of abdominal fat distribution in lean women with polycystic ovary syndrome. Acta Obstet Gynecol Scand 90: 600–608. 21401530 10.1111/j.1600-0412.2011.01124.x

[62] Holte J, Bergh T, Berne C, Berglund L, Lithell H (1994) Enhanced early insulin response to glucose in relation to insulin resistance in women with polycystic ovary syndrome and normal glucose tolerance. J Clin Endocrinol Metab 78: 1052–1058. 8175959 10.1210/jcem.78.5.8175959

[63] Carmina E, Bucchieri S, Mansueto P, Rini G, Ferin M, et al (2009) Circulating levels of adipose products and differences in fat distribution in the ovulatory and anovulatory phenotypes of polycystic ovary syndrome. Fertil Steril 91: 1332–1335. 18455165 10.1016/j.fertnstert.2008.03.007

[64] Moran L, Teede H (2009) Metabolic features of the reproductive phenotypes of polycystic ovary syndrome. Hum Reprod Update 15: 477–488. 19279045 10.1093/humupd/dmp008

[65] Kuchenbecker WK, Groen H, van Asselt SJ, Bolster JH, Zwerver J, et al (2011) In women with polycystic ovary syndrome and obesity, loss of intra-abdominal fat is associated with resumption of ovulation. Hum Reprod 26: 2505–2512. 21771766 10.1093/humrep/der229

[66] Dolfing JG, Stassen CM, van Haard PM, Wolffenbuttel BH, Schweitzer DH (2011) Comparison of MRI-assessed body fat content between lean women with polycystic ovary syndrome (PCOS) and matched controls: less visceral fat with PCOS. Hum Reprod 26: 1495–1500. 21406446 10.1093/humrep/der070

[67] Barber TM, Golding SJ, Alvey C, Wass JA, Karpe F, et al (2008) Global adiposity rather than abnormal regional fat distribution characterizes women with polycystic ovary syndrome. J Clin Endocrinol Metab 93: 999–1004. 18089693 10.1210/jc.2007-2117

[68] Kuchenbecker WK, Groen H, Zijlstra TM, Bolster JH, Slart RH, et al (2010) The subcutaneous abdominal fat and not the intraabdominal fat compartment is associated with anovulation in women with obesity and infertility. J Clin Endocrinol Metab 95: 2107–2112. 20200335 10.1210/jc.2009-1915

[69] Roland AV, Nunemaker CS, Keller SR, Moenter SM (2010) Prenatal androgen exposure programs metabolic dysfunction in female mice. Journal of Endocrinology 207: 213–223. 20713501 10.1677/JOE-10-0217 PMC3612271

[70] Friedman JM, Halaas JL (1998) Leptin and the regulation of body weight in mammals. Nature 395: 763–770. 9796811 10.1038/27376

[71] Koch L, Wunderlich FT, Seibler J, Konner AC, Hampel B, et al (2008) Central insulin action regulates peripheral glucose and fat metabolism in mice. J Clin Invest 118: 2132–2147. 18451994 10.1172/JCI31073 PMC2350427

[72] Chazenbalk G, Bertolotto C, Heneidi S, Jumabay M, Trivax B, et al (2011) Novel pathway of adipogenesis through cross-talk between adipose tissue macrophages, adipose stem cells and adipocytes: evidence of cell plasticity. PLoS One 6: e17834. 21483855 10.1371/journal.pone.0017834 PMC3069035

[73] Gottfried E, Kunz-Schughart LA, Weber A, Rehli M, Peuker A, et al (2008) Expression of CD68 in non-myeloid cell types. Scandinavian Journal of Immunology 67: 453–463. 18405323 10.1111/j.1365-3083.2008.02091.x

[74] Cousin B, Munoz O, Andre M, Fontanilles AM, Dani C, et al (1999) A role for preadipocytes as macrophage-like cells. Faseb Journal 13: 305–312. 9973318 10.1096/fasebj.13.2.305

[75] Beranek JT (2005) CD68 is not a macrophage-specific antigen. Annals of the Rheumatic Diseases 64: 342–343. 15647451 PMC1755344

[76] Kunisch E, Fuhrmann R, Roth A, Winter R, Lungershausen W, et al (2004) Macrophage specificity of three anti-CD68 monoclonal antibodies (KP1, EBM11, and PGM1) widely used for immunohistochemistry and flow cytometry. Annals of the Rheumatic Diseases 63: 774–784. 15194571 10.1136/ard.2003.013029 PMC1755048

[77] Avram MM, Avram AS, James WD (2007) Subcutaneous fat in normal and diseased states - 3. Adipogenesis: From stem cell to fat cell. Journal of the American Academy of Dermatology 56: 472–492. 17317490 10.1016/j.jaad.2006.06.022

[78] Gonzalez F (2012) Inflammation in Polycystic Ovary Syndrome: Underpinning of insulin resistance and ovarian dysfunction. Steroids 77: 300–305. 22178787 10.1016/j.steroids.2011.12.003 PMC3309040

[79] Piotrowski PC, Rzepczynska IJ, Kwintkiewicz J, Duleba AJ (2005) Oxidative stress induces expression of CYP11A, CYP17, star and 3 beta HSD in rat theca-interstitial cells. Journal of the Society for Gynecologic Investigation 12: 319a–319a.

[80] Ortega I, Stener-Victorin E, Villanueva JA, Sokalska A, Stanley SD, et al (2011) Letrozole Increases Growth of Rat Theca-Interstitial Cells and Cyp17a1 Gene Expression in the Rat Ovary. Fertility and Sterility 96: S114–S114. 10.1016/j.fertnstert.2012.11.006 PMC3667963 23200686

[81] Spaczynski RZ, Arici A, Duleba AJ (1999) Tumour necrosis factor-alpha stimulates proliferation of rat ovarian theca-interstitial cells. Biology of Reproduction 61: 993–998. 10491635 10.1095/biolreprod61.4.993

[82] Spaczynski RZ, Arici A, Duleba AJ (1999) Tumor necrosis factor-alpha stimulates proliferation of rat ovarian theca-interstitial cells. Biol Reprod 61: 993–998. 10491635 10.1095/biolreprod61.4.993

[83] Townson DH, Liptak AR (2003) Chemokines in the corpus luteum: implications of leukocyte chemotaxis. Reprod Biol Endocrinol 1: 94. 14613530 10.1186/1477-7827-1-94 PMC293429

[84] Liu Z, de Matos DG, Fan HY, Shimada M, Palmer S, et al (2009) Interleukin-6: an autocrine regulator of the mouse cumulus cell-oocyte complex expansion process. Endocrinology 150: 3360–3368. 19299453 10.1210/en.2008-1532 PMC2703543

[85] Ehrmann DA, Barnes RB, Rosenfield RL, Cavaghan MK, Imperial J (1999) Prevalence of impaired glucose tolerance and diabetes in women with polycystic ovary syndrome. Diabetes Care 22: 141–146. 10333916 10.2337/diacare.22.1.141

[86] Norman RJ, Masters L, Milner CR, Wang JX, Davies MJ (2001) Relative risk of conversion from normoglycaemia to impaired glucose tolerance or non-insulin dependent diabetes mellitus in polycystic ovarian syndrome. Human Reproduction 16: 1995–1998. 11527911 10.1093/humrep/16.9.1995

[87] Bellamy L, Casas JP, Hingorani AD, Williams D (2009) Type 2 diabetes mellitus after gestational diabetes: a systematic review and meta-analysis. Lancet 373: 1773–1779. 19465232 10.1016/S0140-6736(09)60731-5

[88] Toulis KA, Goulis DG, Kolibianakis EM, Venetis CA, Tarlatzis BC, et al (2009) Risk of gestational diabetes mellitus in women with polycystic ovary syndrome: a systematic review and a meta-analysis. Fertility and Sterility 92: 667–677. 18710713 10.1016/j.fertnstert.2008.06.045

[89] Devlieger R, Casteels K, Van Assche F (2008) Reduced adaptation of the pancreatic B cells during pregnancy is the major causal factor for gestational diabetes: Current knowledge and metabolic effects on the offspring. Acta Obstetricia Et Gynecologica Scandinavica 87: 1266–1270. 18846453 10.1080/00016340802443863

[90] Hunt KJ, Schuller KL (2007) The increasing prevalence of diabetes in pregnancy. Obstet Gynecol Clin North Am 34: : 173–199, vii. 10.1016/j.ogc.2007.03.00 PMC2043158 17572266

[91] Metzger BE, Gabbe SG, Persson B, Buchanan TA, Catalano PA, et al (2010) International association of diabetes and pregnancy study groups recommendations on the diagnosis and classification of hyperglycemia in pregnancy. Diabetes Care 33: 676–682. 20190296 10.2337/dc09-1848 PMC2827530

